# Therapeutic efficacy of a combined sage and bitter apple phytopharmaceutical in chronic DSS-induced colitis

**DOI:** 10.1038/s41598-017-13985-x

**Published:** 2017-10-27

**Authors:** Maximilian Hoffmann, Ulla Schwertassek, Aleksandra Seydel, Klaus Weber, Sunna Hauschildt, Jörg Lehmann

**Affiliations:** 1 Fraunhofer Institute for Cell Therapy and Immunology, Department Therapy Validation, Leipzig, Germany; 2 AnaPath GmbH, Liestal, Switzerland; 3 Faculty of Biological Sciences, Pharmacy, and Psychology, University of Leipzig, Department of Immunobiology, Leipzig, Germany

## Abstract

Inflammatory bowel diseases are multifactorial disorders of the gastrointestinal tract with rising incidence worldwide. Current standard therapies are only partially effective and often show severe adverse effects. Thus, novel, more efficient and well-tolerated therapeutic options are urgently needed. We have studied the therapeutic potential of a phytopharmaceutical combining sage and bitter apple (SBA) in the mouse model of chronic dextran sulfate sodium (DSS) colitis. SBA represents a traditional medicine against diarrhea and was shown to exhibit anti-inflammatory effects *in vitro*. In the chronic DSS colitis model SBA treatment significantly reduced clinical symptoms in a dose-dependent manner. The positive therapeutic effect of SBA was characterized by a decreased histopathological score indicating tissue healing. Moreover, the number of neutrophils as well as the expression of the neutrophil-recruiting chemokine CXCL-1/KC in the colon tissue was significantly reduced, whereas the recruitment of macrophages was induced. Also, the expression of inflammatory markers was significantly suppressed, while the expression of the anti-inflammatory cytokine interleukin-10 was induced in colon tissue following treatment with SBA. Phytopharmaceuticals are increasingly recognized as potential therapeutics in IBD. Thus, based on the results from this study, SBA can be considered as an alternative or supplementary option for IBD therapy.

## Introduction

Inflammatory bowel diseases (IBD), such as Crohn’s disease (CD) and ulcerative colitis (UC), are chronic-relapsing inflammatory disorders of the gastrointestinal tract. Due to chronic inflammation with ensuing disintegration of the gut epithelium and the underlying tissue, patients present with symptoms such as abdominal pain, diarrhea, bloody stool and weight loss^1–3^. IBD are multifactorial diseases with unknown etiology and increasing prevalence and incidence, particularly in countries with a western lifestyle^4^. Pharmacological and surgical interventions are the two main approaches in treatment of IBD. The most commonly used drugs for IBD treatment are aminosalicylates, glucocorticoids, and immunomodulators which are used to reduce or inhibit the chronic inflammatory response. However, these drugs show limited efficiency with respect to long-term remission and are associated with severe side effects^5^. Antibodies inhibiting tumor necrosis factor (TNF)-α, such as infliximab or adalimumab, have shown clinical efficacy but caused serious side effects and are relatively expensive^6,7^. Furthermore, 20–40% of patients do not respond to anti-TNF treatment (primary failure) and up to 50% lose their effective therapeutic response within 1 to 2 years (secondary failure)^8–10^. Surgery is needed for 20–30% of patients who are non-responders and develop complications such as perforation, refractory rectal bleeding or toxic megacolon. Even after resection, patients suffer from postoperative complications^3,11^. Therefore, there is an urgent need for new therapeutic options that are well-tolerated and improve clinical symptoms as well as initiate tissue healing.

In recent years, the use of phytopharmaceuticals has come into focus as supplementary therapy or in combination therapies with classical IBD therapeutics. Several phytosubstances have already been tested in different IBD models and revealed promising therapeutic effects^12–15^. Sage (*Salvia officinalis* L.) is an oil-producing subshrub that has long been cultured for culinary use. Furthermore, sage is known for its therapeutic effects, including anti-microbial, anti-viral, anti-inflammatory, and pain-relieving properties that are due to the antioxidant as well as anti-inflammatory activity of different flavonoids^16–21^. In contrast, the desert plant bitter apple (*Citrullus colocynthis* (L.) SCHRAD.) containing curcubitacins has been used for centuries as a therapeutic purgative, but has also shown anti-diarrheal effects in combination with other substances^22,23^. Furthermore, anti-microbial, anti-fungal, anti-inflammatory, and analgesic effects have been described in context with the medical use of bitter apple^24,25^.

Various animal models are available to study pathogenesis of IBD or to prove potentially new therapeutic approaches. The acute dextran sulfate sodium (DSS)-induced colitis model is the most commonly used mouse model in IBD research. However, its major caveats are the lack of a chronic adaptive immune response as well as severe body weight loss of the animals. Therefore, our group has developed a refined mouse model of chronic DSS-induced colitis that reflects clinical symptoms of IBD without risky weight loss usually observed in DSS colitis models [Hoffmann *et al*., submitted]. In this model, the standard-of-care (SoC) therapeutics 6-thioguanine (6-TG) and cyclosporine A (CsA) reduced both clinical and histopathological symptoms, qualifying this model for testing potentially new IBD therapeutics. In the current study, we investigated the therapeutic effect of a traditional phytopharmaceutical extract based on sage and bitter apple (SBA) in the chronic DSS colitis model with respect to reduction of clinical symptoms and histopathology as well as its impact on immune cell infiltration into the colon tissue.

## Results

### SBA reduced clinical symptoms in chronic DSS colitis

**Figure 1:**
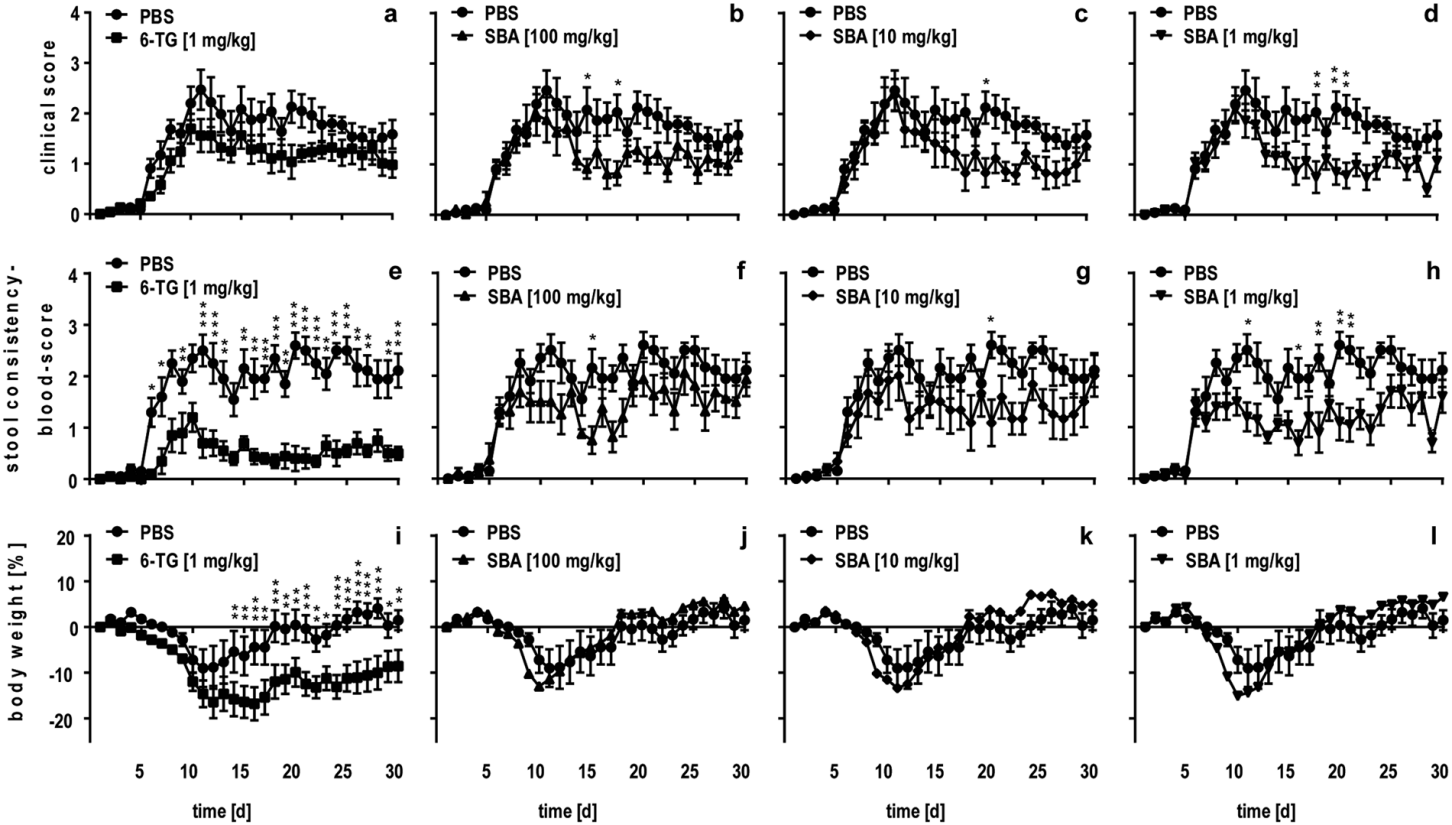
SBA reduced clinical symptoms in chronic DSS colitis. Chronic colitis was induced in BALB/c mice by feeding of 2% DSS in drinking water for 7 days followed by 10 days of 1% DSS and another 12 days of 2% DSS. Animals were treated from days 1–10 and 18–22 with 6-TG (1 mg/kg) as positive control (**a**,**e**,**i**) or at days 1, 3, 5, 7, 9, 18, 20, 22 with different concentrations of SBA: 100 mg/kg (**b**,**f**,**j**); 10 mg/kg (**c**,**g**,**k**); or 1 mg/kg (**d**,**h**,**l**). Animals treated orally with PBS from days 1-10 and 18–22 were used as vehicle control. Clinical score (**a**–**d**), stool-consistency-blood score (**e**–**h**), and body weight (**i**–**l**) were evaluated daily and are shown as mean ± SEM for 6–10 animals per group. Statistics: Two-way ANOVA followed by a Bonferroni’s multiple comparisons test. *P < 0.05; **P < 0.01; ***P < 0.001 versus vehicle control group.

To prove whether SBA was of potential therapeutic use in IBD therapy, the extract was tested in the refined chronic DSS colitis model recently established by our group. Animals were treated with either different doses of SBA at days 1, 3, 5, 7, 9, 18, 20, 22 or with 6-TG (1 mg/kg) as positive treatment control from days 1-10 and 18-22. The unequal treatment protocols of SBA and 6-TG are the result of individual optimization of both therapeutic approaches. A clinical score based on body weight loss, stool consistency and colonic hemorrhage was determined daily (Fig. 1a–d). In addition, a stool-consistency-blood score that focused on the clinically most relevant symptoms of diarrhea and colonic hemorrhage was evaluated (Fig. 1e–h). Clinical symptoms increased rapidly starting from day 5 and reached a peak on days 10-11. In the vehicle control group (PBS), clinical symptoms persisted on a constant level throughout the experiment. Treatment with the positive control, 6-TG, ameliorated overall clinical symptoms moderately (Fig. 1a) but had a significant therapeutic effect on the development of diarrhea and colonic hemorrhage (Fig. 1e). All concentrations of SBA showed a modest therapeutic effect with respect to the clinical score (Fig. 1b–d) as well as the stool-consistency-blood score (Fig. 1f–h). Interestingly, the lowest concentration of 1 mg/kg induced the most pronounced clinical effects that were partly significant if compared to the vehicle control (Fig. 1d and h). The differences between clinical and stool-consistency-blood score was due to the changes in body weight (Fig. 1i–l). In our refined chronic DSS colitis model, body weight loss of approximately 10–15% is initially observed when clinical symptoms start to develop around day 5. In contrast to other DSS colitis models, the body weight increases again during the chronification phase and stabilizes until the end of the experiment without amelioration of other clinical symptoms, i.e. diarrhea and colonic hemorrhage. Thus, the increased body weight loss after treatment with 6-TG (Fig. 1i) accounted for the seemingly reduced efficacy of the SoC in the clinical score (Fig. 1a). However, with respect to the stool-consistency-blood score, a clear therapeutic effect could be observed indicating that 6-TG significantly ameliorated diarrhea and colonic hemorrhage (Fig. 1e).

### SBA reduced the inflammatory reaction in the colon tissue

**Figure 2:**
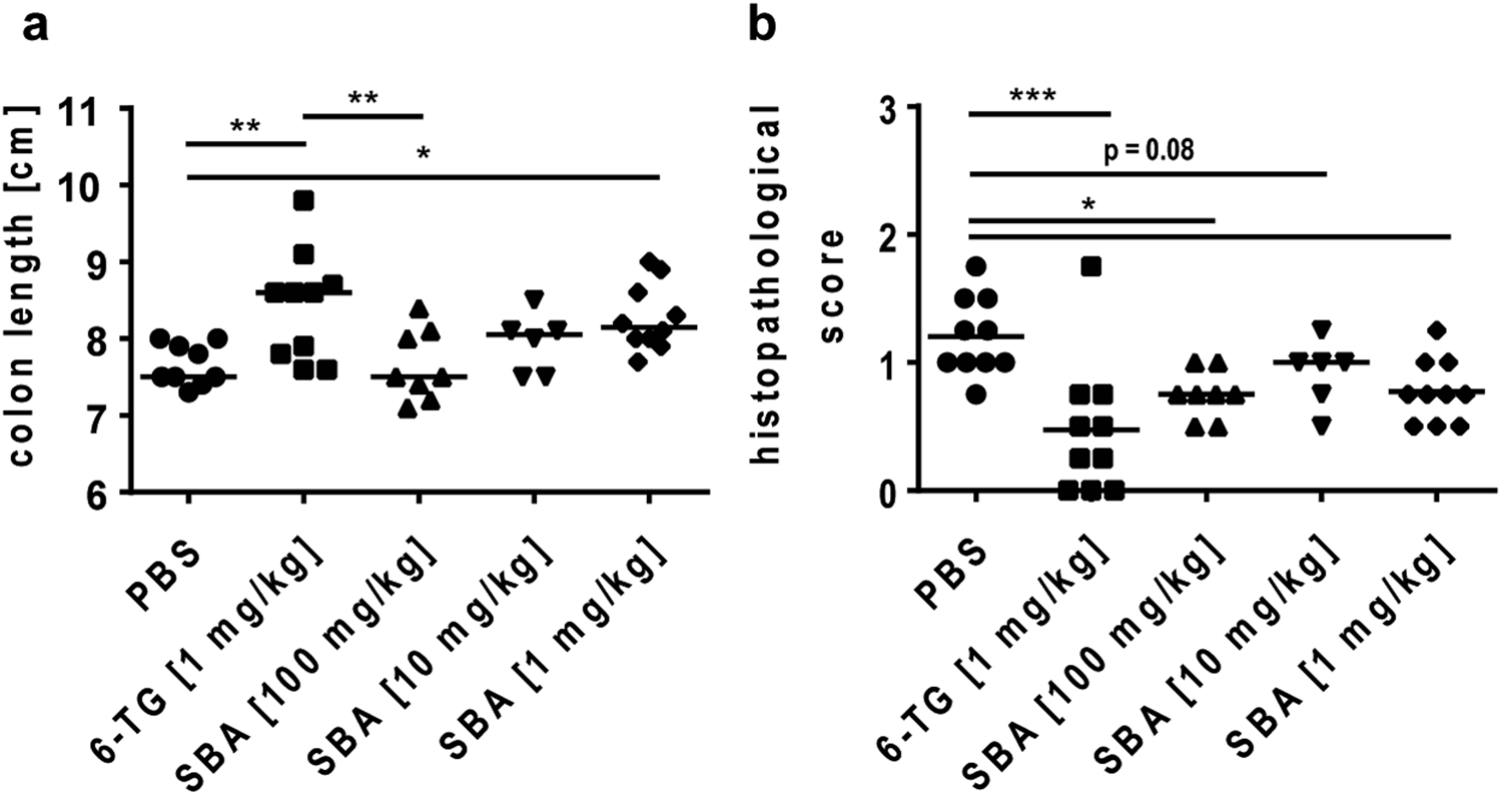
SBA ameliorated pathology of the colon tissue. Chronic colitis was induced in BALB/c mice by feeding of 2% DSS in drinking water for 7 days followed by 10 days of 1% DSS and another 12 days of 2% DSS. Animals were treated orally from days 1–10 and 18–22 with 6-TG (1 mg/kg) as positive control or at days 1, 3, 5, 7, 9, 18, 20, 22 with different concentrations of SBA: 100 mg/kg; 10 mg/kg; or 1 mg/kg. Animals treated orally with PBS from days 1–10 and 18–22 were used as vehicle control. The colon length was determined as a marker for the extent of tissue inflammation and fibrosis (**a**). The histopathological score was determined based on hematoxylin-eosin-stained cross sections of the distal colon (**b**). The median of individual data points is indicated. Statistics: One-Way ANOVA with Dunnett’s multiple comparison test; number of animals per group = 6–10; *p < 0.05; **p < 0.01 versus vehicle control group.

For histopathological evaluation, the whole colon was dissected on day 30 and its length was measured as a parameter for the extent of colonic inflammation (Fig. 2a). In DSS colitis models, shortening of the colon is frequently observed due to inflammation-induced fibrosis and consequent scarring of the colon tissue. As expected from the clinical outcome, mice treated with the SoC control (6-TG) had a significantly longer colon than mice treated with the vehicle control (PBS). Interestingly, treatment with the lowest dose of SBA also resulted in a significantly longer colon if compared to the vehicle control whereas the higher doses of SBA did not show significant therapeutic effects. Based on hematoxylin-eosin-stained cross sections of the distal colon, a histopathological score was evaluated including the assessment of infiltrating lymphocytes, granulocytes, and macrophages as well as the degree of edema and superficial necrosis (Fig. 2b). Reflecting the results obtained for the clinical symptoms, 6-TG-treated animals showed the lowest signs of inflammation with a minimum number of infiltrating granulocytes. Likewise, treatment with the high- and low-dose SBA resulted in a significant reduction of the histopathological score. In contrast to the clinical symptoms, the histopathological signs of inflammation were also reduced by the highest dose of SBA, which was due to a lower number of infiltrating lymphocytes in the colonic tissue of these animals.

**Figure 3:**
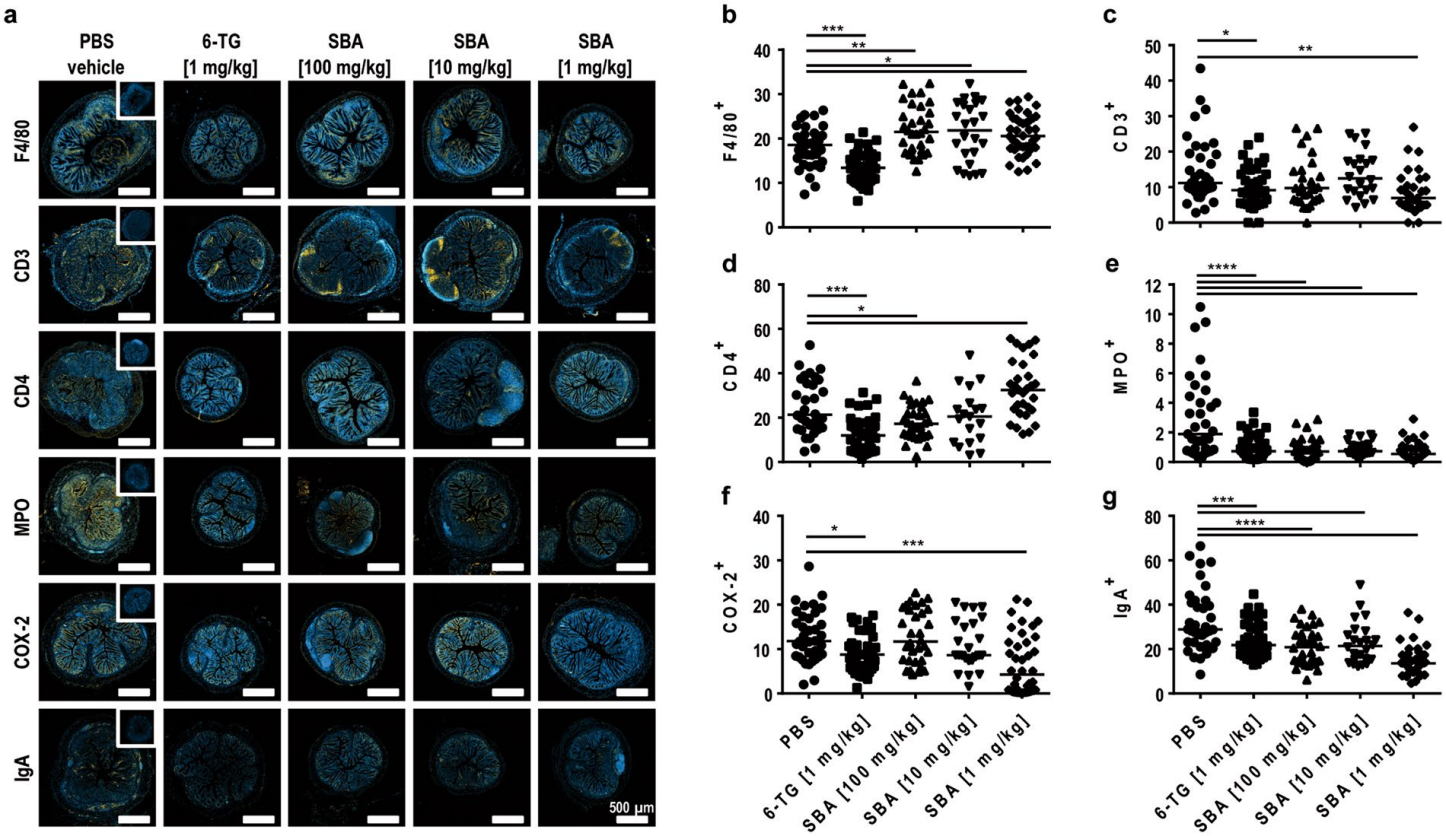
SBA altered infiltration of inflammatory cells into the colon tissue. Chronic colitis was induced in BALB/c mice by feeding of 2% DSS in drinking water for 7 days followed by 10 days of 1% DSS and another 12 days of 2% DSS. Animals were treated orally from days 1–10 and 18–22 with 6-TG as positive control (1 mg/kg) or at days 1, 3, 5, 7, 9, 18, 20, 22 with different concentrations of SBA: 100 mg/kg; 10 mg/kg; or 1 mg/kg. Animals treated orally with PBS from days 1–10 and 18–22 were used as vehicle control. Cross sections of the distal colon were stained for the macrophage marker F4/80, the T cell markers CD3 and CD4, the inflammatory markers COX-2 and MPO, and for IgA; scale bars: 500 µm (**a**). Stained cross sections (2 sections/mouse) were digitized with a slide scanner (Zeiss AxioScan.Z1) and expression was quantified using the ImageJ software program and is shown as marker-positive area relative to the DAPI-positive area (**b**–**g**). The median of individual data points is indicated. Statistics: One-Way ANOVA with Dunnett’s multiple comparison test; number of animals per group = 6–10; *p < 0.05; **p < 0.01; ***p < 0.001; ****p < 0.0001 versus vehicle control group.

To characterize infiltrating immune cells in more detail, cross sections of the distal colon were stained for macrophages (F4/80), T cells (cluster of differentiation (CD) 3), T helper (Th) cells (CD4), as well as the inflammatory markers myeloperoxidase (MPO), cyclooxygenase 2 (COX-2), and immunoglobulin A (IgA) (Fig. 3a). Since a significant contribution of cytotoxic T cells was never observed in the refined chronic DSS model (unpublished data), we refrained from staining tissue sections for CD8 expression. Digital photomicrographs of stained cross sections were taken by a slide scanner and quantified as area positive for the respective antigen relative to the area of 4′,6-Diamidin-2-phenylindol (DAPI)-positive nuclei (Fig. 3b–g). F4/80, a typical surface marker for murine macrophages, was significantly reduced in the cross sections from 6-TG-treated animals if compared to the vehicle control group (Fig. 3b). Interestingly, F4/80 expression was significantly increased in all SBA-treated animals, indicating that SBA induced recruitment of macrophages to the inflamed tissue. Quantification of CD3-positive cells (predominantly representing T cells) revealed a significantly decreased number of T cells in 6-TG-treated animals and in the treatment group receiving low-dose SBA (Fig. 3c). To characterize T cells in more detail, colon sections were also stained for CD4-positive Th cells and marker expression was quantified. Consistent with the result for CD3, administration of 6-TG caused a significant reduction of Th cells if compared to PBS-treated animals (Fig. 3d). Treatment with SBA resulted in a dose-dependent response: Animals receiving the highest dose of SBA showed a decrease whereas the lowest dose induced an increase of CD4-positive Th  cells if compared to the vehicle control group. Myeloperoxidase, an enzyme produced by neutrophils and monocytes, is commonly upregulated during an inflammatory response and is responsible for the regulation and termination of inflammatory processes. In colon tissue sections, MPO expression was significantly reduced by all therapeutic treatments if compared to PBS-treated animals indicating that neutrophil and monocyte number and/or activity was reduced by all tested therapeutics (Fig. 3e). The expression of the inflammatory marker COX-2 is usually increased during inflammatory responses. In the colon tissue of 6-TG-treated animals, a significant reduction of Cox-2 expression was observed (Fig. 3f). The lowest dose of SBA induced an even more pronounced therapeutic effect whereas the higher doses of SBA showed reduced effects which were dose-dependent but not significant. IgA is produced by mucosal tissue plasma cells located close to the epithelial barrier and is also secreted into the mucus and the gut lumen. Staining of IgA in the colon tissue demonstrated a strong influence of SBA on this inflammatory marker. All tested doses of the phytoextract were capable of decreasing the amount of IgA in tissue cross sections, with the lowest dose of SBA showing the strongest effect (Fig. 3g).

### SBA restored number but not function of goblet cells

**Figure 4:**
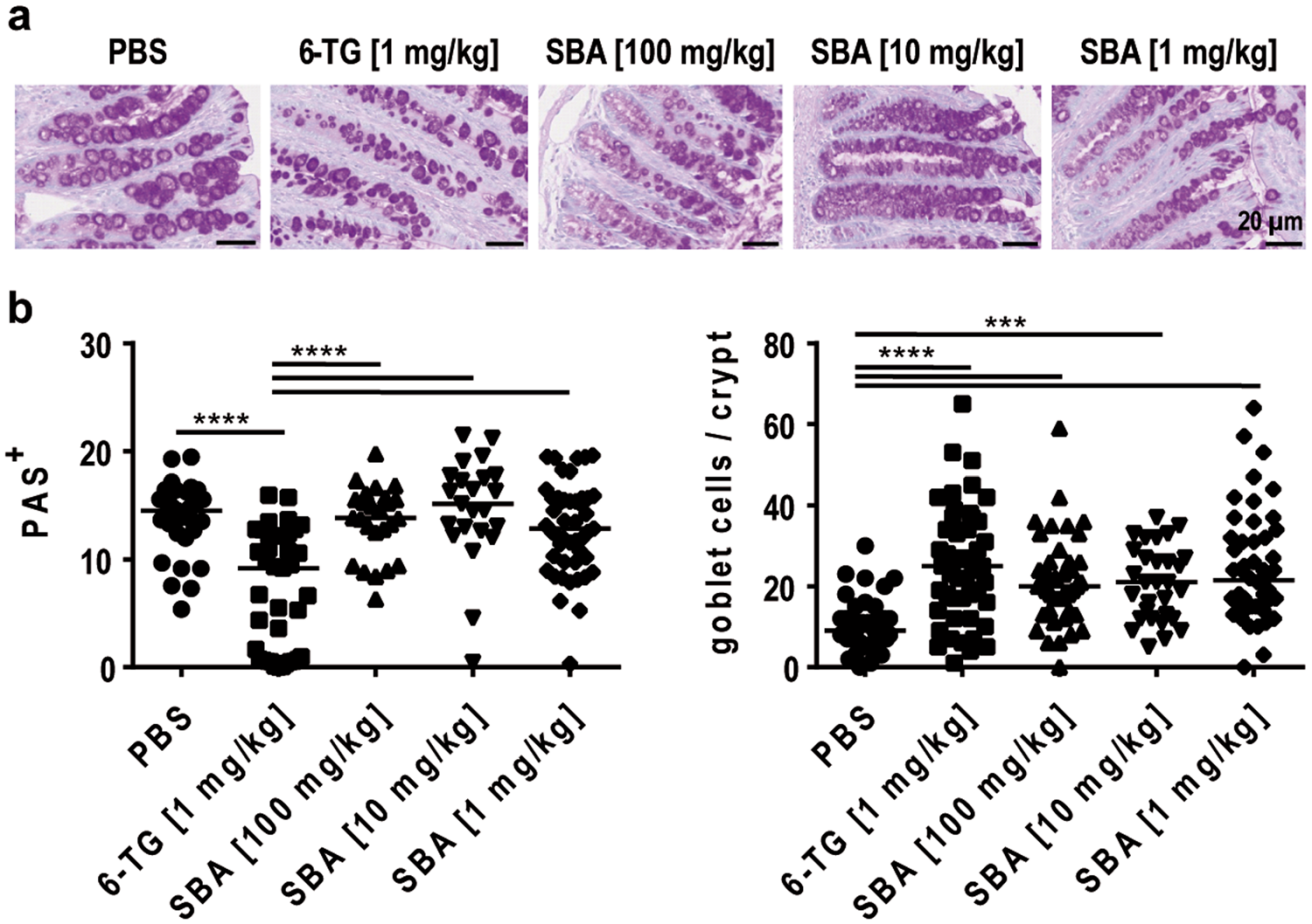
SBA restored number but not function of goblet cells. Chronic colitis was induced in BALB/c mice by feeding of 2% DSS in drinking water for 7 days followed by 10 days of 1% DSS and another 12 days of 2% DSS. Animals were treated orally from days 1–10 and 18–22 with 6-TG as positive control (1 mg/kg) or at days 1, 3, 5, 7, 9, 18, 20, 22 with different concentrations of SBA: 100 mg/kg; 10 mg/kg; or 1 mg/kg. Animals treated orally with PBS from days 1–10 and 18–22 were used as vehicle control. Cross sections of the distal colon were stained with periodic-acid-Schiff reagent and counterstained with hematoxylin; scale bars: 20 µm (**a**). Stained cross sections (2 sections/mouse) were digitized with a slide scanner (Zeiss AxioScan.Z1) and expression was quantified using the ImageJ software program and is shown as PAS-positive area relative to the DAPI-positive area (**b**). The number of goblet cells per crypt was counted manually (**c**). The median of individual data points is indicated. Statistics: One-Way ANOVA with Dunnett’s multiple comparison test; number of animals per group = 6–10; ***P < 0.001; ****P < 0.0001 versus vehicle control group.

To analyze the effect of SBA administration on the production of mucus and the integrity of mucus-producing goblet cells, cross sections of the colon tissue were stained with periodic-acid-Schiff (PAS) reagent and the PAS-positive area was quantified (Fig. 4). Goblet cells are particularly sensitive to DSS treatment and typically change in number, morphology, and capacity to produce mucus in most DSS colitis models. Two contrary observations in terms of goblet cells can be observed in colonic cross sections of DSS-treated animals: A total loss of goblet cells caused by massive ulceration as well as a reactive overproduction of mucus. In the chronic DSS colitis model used in this study, reactive overproduction of mucus is commonly more pronounced resulting in increased PAS staining in DSS-treated animals. Consistently, a significantly reduced PAS staining was observed in colon sections of 6-TG-treated animals (Fig. 4a and b). In contrast, SBA treatment did not show any effect on mucus production if compared to the vehicle control group. In addition to mucus production, the number of goblet cells per crypt was analyzed to determine the effect of treatments on goblet cell integrity (Fig. 4c). Contrary to mucus production, the number of goblet cells per crypt significantly increased after treatment with 6-TG or any dose of SBA. Taken together, these results indicate that 6-TG treatment results in restoration of both mucus production and the integrity of goblet cells in this chronic DSS colitis model. SBA treatment had a positive effect on the number of goblet cells but was not able to efficiently restore normal goblet cell function, i.e. mucus production.

### SBA restored expression of tight junction proteins

**Figure 5:**
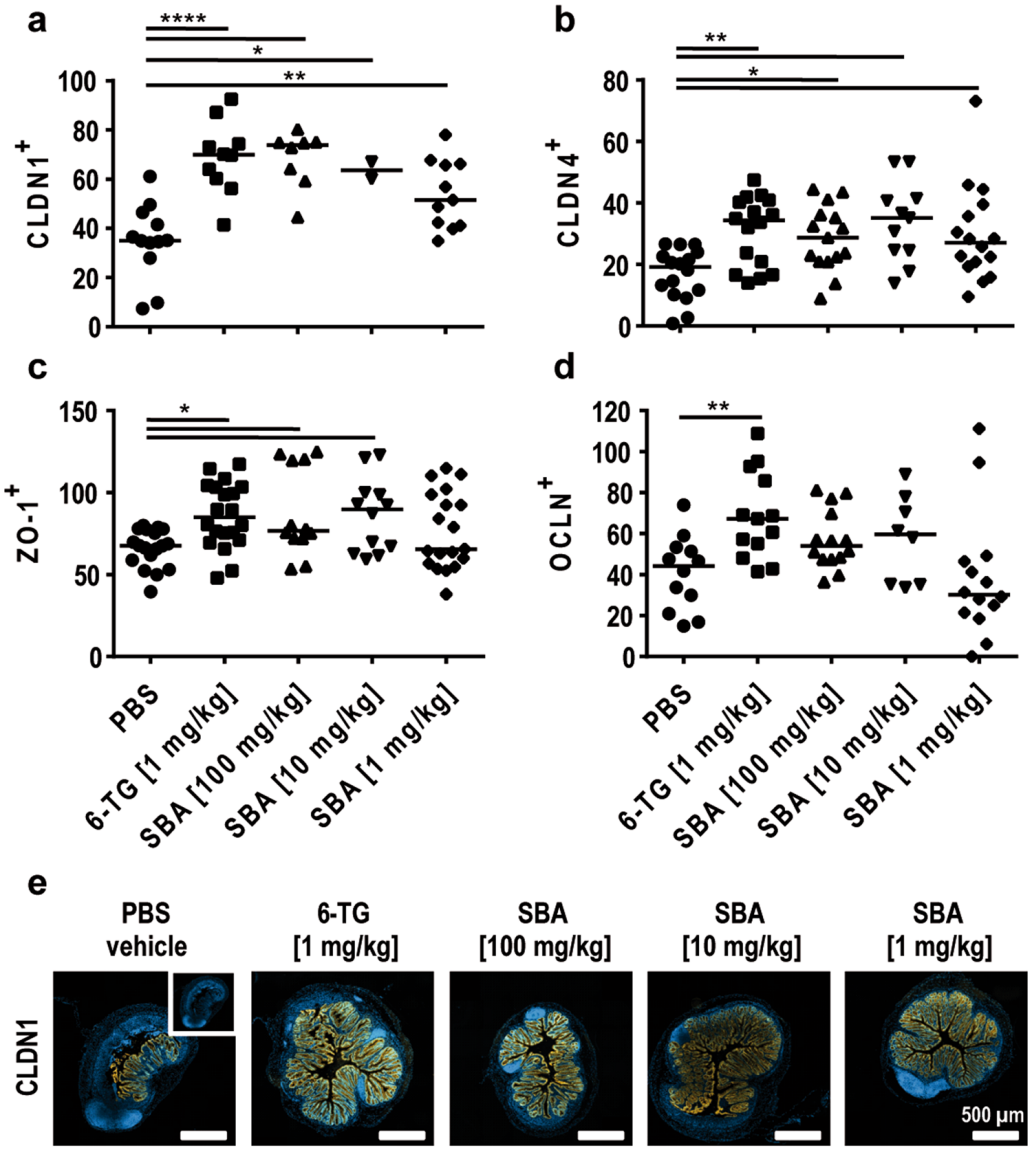
SBA restored expression of tight junction proteins. Chronic colitis was induced in BALB/c mice by feeding of 2% DSS in drinking water for 7 days followed by 10 days of 1% DSS and another 12 days of 2% DSS. Animals were treated orally from days 1–10 and 18–22 with 6-TG as positive control (1 mg/kg) or at days 1, 3, 5, 7, 9, 18, 20, 22 with different concentrations of SBA: 100 mg/kg; 10 mg/kg; or 1 mg/kg. Animals treated orally with PBS from days 1–10 and 18–22 were used as vehicle control. Cross sections of the distal colon were stained for claudin (CLDN)1 (**a**) and CLDN4 (**b**), *Zonula occludens* protein (ZO)-1 (**c**) and occludin (OCLN) (**d**). Stained cross sections (2 sections/mouse) were digitized with a slide scanner (Zeiss AxioScan.Z1) and expression was quantified using ImageJ software program and is shown as TJ protein-positive area relative to the DAPI-positive area. The median of individual data points is indicated. Statistics: One-Way ANOVA with Dunnett’s multiple comparison test; number of animals per group = 1–10; *P < 0.05; **P < 0.01; ****P < 0.0001 versus vehicle control group. Representative images of CLDN1 expression in the colon tissue; scale bars: 500 µm (**e**).

Tight junctions (TJ) are a main structural component of the epithelial layer and thus TJ integrity represents an essential feature of the first defense barrier of the gut. To further characterize the impact of therapeutic treatment on gut epithelial integrity, the expression of TJ proteins Claudin (CLDN) 1 and 4, *Zonula occludens* protein (ZO)-1, and Occludin (OCLN) was determined in cross sections of the colon tissue. DSS treatment is known to disintegrate the epithelial barrier and to compromise the integrity of TJs. Administration of 6-TG significantly induced the expression of all TJ proteins analyzed if compared to the vehicle control group (Fig. 5a–d). Except for OCLN, SBA treatment similarly induced the expression of TJ proteins with the strongest effect observed for CLDN1 (Fig. 5a and e).

### SBA reduced expression of inflammatory markers in the colon tissue

**Figure 6:**
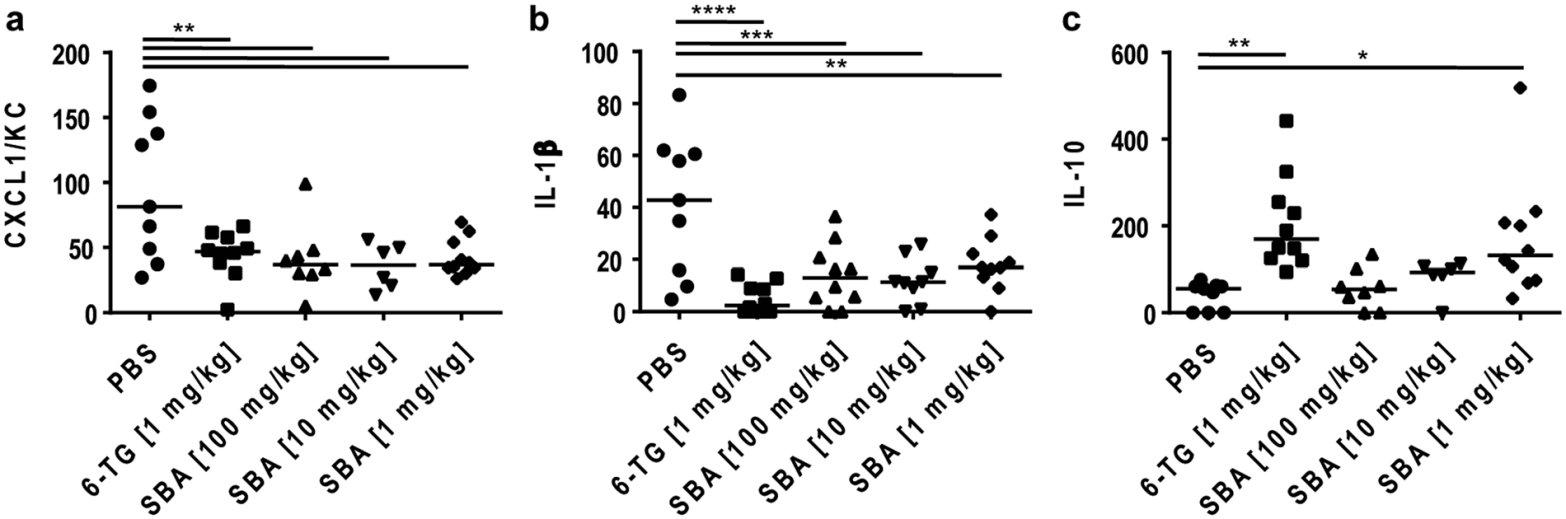
SBA reduced expression of inflammatory markers in the colon tissue. Chronic colitis was induced in BALB/c mice by feeding of 2**%** DSS in drinking water for 7 days followed by 10 days of 1**%** DSS and another 12 days of 2**%** DSS. Animals were treated orally from days 1–10 and 18–22 with 6-TG as positive control (1 mg/kg) or at days 1, 3, 5, 7, 9, 18, 20, 22 with different concentrations of SBA: 100 mg/kg; 10 mg/kg; or 1 mg/kg. Animals treated orally with PBS from days 1–10 and 18–22 were used as vehicle control. Concentrations of CXCL1/KC (**a**), IL-1β (**b**) and IL-10 (**c**) were determined in the supernatant of homogenized tissue samples of the distal colon by ELISA. The median of individual data points is indicated. Statistics: One-Way ANOVA with Dunnett’s multiple comparison test; number of animals per group = 6–10; *p < 0.05; **p < 0.01; ***p < 0.001; ****p < 0.0001 versus vehicle control group.

To determine the expression of soluble inflammatory markers in the colon tissue, a colon homogenate was analyzed for expression of the chemokine (C-X-C motif) ligand 1/KC (CXCL1/KC), the pro-inflammatory cytokine interleukin (IL)-1β, and the anti-inflammatory cytokine IL-10 (Fig. 6). CXCL1/KC is mainly produced by neutrophils and its expression was significantly reduced by all treatments if compared to the vehicle control group (Fig. 6a). IL-1β is known to induce expression of COX-2, cortisone, and IL-6 during inflammatory responses. Similarly to CXCl1/KC, all tested treatments significantly reduced the concentration of IL-1β in the colon tissue homogenate (Fig. 6b), with no dose-dependent differences being observed for SBA treatment. In contrast to the effects on pro-inflammatory markers, SBA treatment induced a dose-dependent increase of IL-10 expression in the colon tissue, with the lowest dose of SBA showing a significant therapeutic effect (Fig. 6c).

## Discussion

Phytochemicals have recently come into focus as potential therapies for the treatment of IBD^26,27^. There is a great interest to harness the beneficial therapeutic effects of these substances and thereby reduce or circumvent the often deleterious adverse effects of conventional therapies. In this study, the potential efficacy of a sage and bitter apple extract (SBA) was proved in a chronic DSS colitis model reflecting the most relevant clinical symptoms of IBD, i.e. diarrhea and colonic hemorrhage, without severe weight loss frequently observed in other DSS colitis models [Hoffmann *et al*., submitted]. Since DSS induces the strongest pathology in the distal part of the colon^28^, this study focused on the analysis of colon tissue from the distal part only. It could be demonstrated that SBA was effective as stand-alone therapy in this chronic IBD model and, for some endpoints, revealed similar therapeutic efficacy as the SoC treatment control 6-TG. Not only did oral administration of SBA ameliorate clinical symptoms, such as stool consistency and colonic hemorrhage, it also significantly reduced histopathology as well as the underlying intestinal inflammation. Although previous studies have demonstrated antioxidant activities of sage in different experimental setups^16,29^, to our knowledge this is the first report to demonstrate that a sage-containing extract was effective in ameliorating experimental colitis through a consistent regulation of immune cell infiltration and cytokine expression in the colon tissue.

To analyze the inflammatory response in the colon as well as its regulation by SBA in more detail, colon tissue sections were stained for different immune cell markers and inflammatory mediators, i.e. F4/80 (macrophages), CD3 (T cells), CD4 (Th cells), MPO, COX-2, and IgA. As a first line of defense against potentially harmful microorganisms, e.g. gut bacteria which have entered the subepithelial space of the intestinal tissue, cells of the innate immune system are immediately recruited to contain the infection and sample antigens to trigger the adaptive immune response. Cell types of the innate immune system that are most prominent in the chronic DSS model include macrophages and neutrophilic granulocytes (neutrophils) which are characterized by expression of the cell surface marker F4/80 or the intracellularly expressed or secreted enzyme myeloperoxidase (MPO), respectively. As demonstrated by immunofluorescence staining of F4/80, 6-TG significantly reduced the number of infiltrating macrophages in the colon tissue. In contrast, SBA-treated mice showed a higher number of macrophages infiltrating colon lesions in DSS colitis compared to control animals. Those macrophages which were directly or indirectly recruited by SBA might belong to the regulatory (M2b) or wound-healing macrophage subtype (M2c), supporting anti-inflammatory activity and tissue repair, respectively^30^. This question should be addressed by staining for subtype-specific markers or cytokines in subsequent studies using this model. Own previous *in-vitro* studies, investigating the effect of SBA on the human monocytic cell line THP-1, revealed significantly enhanced secretion of lipopolysaccharide(LPS)-induced IL-6 and more moderately of LPS-induced IL-10 in a dose-dependent fashion, which is in line with the IL-10 result from the present study. However, SBA had different effects on the expression of pro-inflammatory cytokines in THP-1 cells *in vitro*. The LPS-induced secretion of TNF-α was found to be suppressed, whereas IL-1β was found to be induced implying no clear polarization into the inflammatory M1 or the regulatory M2 macrophage subtype (J. Lehmann *et al*., unpublished).

MPO is mainly produced by neutrophils and exerts anti-microbial activity by catalyzing the production of hypochlorous acid during the neutrophil’s respiratory burst^31^. MPO can be detected in the colon tissue as cell-bound and secreted molecule and together with DAPI staining identifies MPO-producing cells. In addition, MPO can be found in the colon crypts and the gut lumen, particularly in DSS-treated animals. Administration of 6-TG as well as SBA (all tested doses) significantly reduced MPO expression in diseased animals. CXCL1/KC is produced by macrophages, neutrophils or epithelial cells and has neutrophil-chemoattractant activity^32,33^. Thus, in addition to MPO expression, the up-regulation of the chemokine CXCL1/KC represents a further endpoint demonstrating neutrophil infiltration. Consistent with the results obtained for MPO staining, analysis of CXCL1/KC in colon homogenate revealed significant reduction of this neutrophil-attractant chemokine after treatment with either 6-TG or SBA.

Dysregulation of T cell homeostasis is considered a major factor in the pathogenesis of IBD. Chronic activation of inflammatory T cells, i.e. Th1, Th2 and Th17 cells, and impaired regulation by Treg cells promote a continuous inflammatory response in the colon^34^. In the chronic colitis model applied in the present study, treatment with 6-TG or with the lowest dose of SBA caused significant reduction of the number of CD3^+^ T cells in colon tissue. Likewise, 6-TG reduced the number of CD4^+^ Th cells in the colon, while SBA showed ambiguous effects depending on the applied dose. The highest applied dose of SBA significantly reduced the number of CD4^+^ Th cells similar to 6-TG. However, the lowest SBA dose even increased the infiltration of Th cells in colon tissue. The reason for these divergent dose-dependent SBA effects remains unclear but let assume different effects of low- and high-dose SBA on the regulation of Th cell differentiation and/or recruitment.

IgA is produced by plasma cells residing in the lamina propria and contributes crucially to barrier function of the gut endothelium as well as homeostasis of the mucosal immune system^35,36^. In our refined chronic DSS colitis model, IgA expression is increased after DSS uptake and can be detected as both cell-bound and secreted IgA in colon tissue. In the present study, SBA administration efficiently reduced IgA expression in the colon, in which the lowest SBA dose was even exceeding the therapeutic effect of the positive control 6-TG. It can thus be speculated that SBA either directly or indirectly regulates the recruitment and/or activation of IgA-producing plasma cells in the mucosal tissue.

Another important barrier mechanism of the gut epithelium is the production of mucus by goblet cells. The mucus barrier can be subdivided into an outer layer that can be penetrated by commensal bacteria and an inner layer that is normally impermeable to the gut microbiota^37^. In chronic DSS colitis, goblet cells are frequently destroyed or functionally impaired and consequently the mucus barrier is significantly disturbed. In the current study, SBA treatment was capable of restoring goblet cell number, indicating a tissue-healing capacity of the phytochemical. Interestingly, colonic mucus production in SBA-treated mice was even increased compared to 6-TG treated animals possibly indicating a functional overreaction of restored goblet cells as a response to the tissue injury induced by DSS treatment. Thus, therapeutic efficacy of SBA was inferior to 6-TG with respect to goblet cell function.

The integrity of the epithelial barrier is primarily mediated by tight junction (TJ) proteins, such as CLDN1, CLDN4, ZO-1 and OCLN. To further analyze the effect of SBA on the integrity of the epithelial barrier, colon tissue sections were analyzed for the aforementioned TJ proteins. Except for OCLN, SBA treatment significantly increased TJ protein expression in colon tissue, with the strongest effect being observed for CLDN1. Together with the reduced histopathological score and the induction of goblet cell regeneration, these results indicate that therapy with SBA has a strong capacity to induce healing processes within the epithelial barrier.

Two main categories have been proposed for the therapeutic effect of phytochemicals in IBD models, i.e. their antioxidant functions as well as their capacity to inhibit pro-inflammatory cytokines and enzymes, such as TNF-α, IL-1β, IL-6, IFN-γ, iNOS, COX-2, and MPO^26^. For example, anti-inflammatory effects are known for different substances contained in sage, such as flavonoids^38–41^. Consistent with this idea, in our study SBA treatment significantly reduced the expression of IL-1β, one of the key cytokines in the induction of inflammatory responses, as well as COX-2 and MPO in colon tissue of DSS-treated animals. Furthermore, SBA induced expression of the anti-inflammatory cytokine IL-10, which is frequently dysregulated in both CD and UC^42^, possibly resulting in suppression of pro-inflammatory cytokines and chemokines as well as inhibition of T cell activation.

In summary, we propose that SBA represents an effective therapeutic agent for the treatment of tissue inflammation in the chronic DSS colitis model by down-regulating different stages of the inflammatory process. In addition, SBA initiates healing of the epithelial layer and regeneration of the crypt structure. Further investigation of SBA and other phytochemicals could reveal a new class of therapeutics for IBD with little side effects to be applied as stand-alone therapy or in combination therapies with SoC treatments.

## Material and Methods

### Animals

Female BALB/c mice (10 weeks old; 20–24 g) were purchased from Janvier (Saint-Berthevin, France). Mice were housed as five animals per cage in a temperature- and light/dark cycle-controlled environment (23 °C, 12 h/12 h light/dark, 50% humidity). They had free access to pelleted standard rodent chow and water *ad libitum*. Animals were acclimatised to the environmental conditions for 14 days before starting of treatment. All experimental procedures were approved by the State Animal Care and Use Committee (Landesdirektion Sachsen, Leipzig, Germany, TVV 38/13) and were carried out in accordance with the European Communities Council Directive (86/609/EEC) for the Care and Use of Laboratory Animals. For organ collection, all animals were sacrificed using carbon dioxide. All efforts were made to minimize suffering of the animals.

### Induction of chronic DSS colitis

Chronic DSS colitis was induced as described recently [Hoffmann *et al*., submitted]. In brief, administration of 2% DSS (MW 36,000–50,000, Lot-No. M7191; MP Biomedicals, Santa Ana, USA) in autoclaved drinking water for 7 days, followed by 1% DSS for 10 days and 2% DSS for another 12 days. DSS solutions were changed every 3 to 4 days. Animals were inspected daily for overall physical and behavioural appearance, and body weight loss as well as scores for stool consistency and blood in stool were assessed. The clinical score was calculated as the average of the scores for body weight loss, stool consistency, and blood in stool. The stool-consistency-blood score was calculated as the average of the scores for stool consistency and blood in stool.

### Drug treatment

Drug solutions or homogenous suspensions were prepared in sterile 0.15 M phosphate-buffered saline (PBS). The following test compounds and doses were used: 6-thioguanine (6-TG) −1 mg/kg (Santa Cruz Biotechnology, Dallas, USA); a combined dry extract from sage (*Salvia officinalis*) and bitter apple (*Citrullus colocynthis*) (SBA) −100, 10 or 1 mg/kg (Bombastus Werke AG, Freital, Germany). The sage flowers were macerated with 68% ethanol in a drug-to-extract ratio of 1: 0.37–0.45. In the bitter apple extract, a drug-to-extract ratio of 1: 3.4–4.3 with 42% ethanol was used. The ratio of sage and bitter apple in the dry extract was 5: 1.

The doses were calculated daily based on the individual body weight. 6-TG (1 mg/kg) was administered by i.p. injection from days 1 to 10 and days 18 to 22 in a volume of 10 ml/kg. SBA was given by oral gavage at days 1, 3, 5, 7, 9, 18, 20 and 22. Control animals received orally PBS only.

### Dissection

On day 30, animals were sacrificed in deep anaesthesia using carbon dioxide. The colon was dissected and the length from caecum to anus was measured. Two parts of 0.3 to 0.5 cm of the most distal colon were taken for histology. The adjacent part (1.0 cm) was taken for enzyme-linked immunosorbent assay (ELISA) analysis.

### Histological analysis

For each mouse, two tissue samples from the distal colon were fixed in 4% buffered formaldehyde for 24 h, dehydrated in grade ethanol, and embedded in paraffin. Tissue sections were cut at 3 µm on a rotary microtome (LEICA RM2255, Nussloch, Germany), mounted on glass slides and dried on a hotplate (60 °C). Sections were cleared, hydrated, and stained with haematoxylin and eosin (H&E) or periodic-acid-Schiff reagent (PAS). Pictures were taken from selected samples and digitized using an image scanner (Zeiss AxioScan.Z1; Zeiss, Jena, Germany). Stained sections were evaluated by a trained pathologist with regard to the extent of inflammation and superficial necrosis, as well as the infiltration of granulocytes, lymphocytes and macrophages into the tissue. For a detailed investigation of goblet cell distribution and mucus production, the amount of goblet cells per crypt was analysed for 5 randomly selected crypts per colon cross section and mouse using PAS-stained cross sections.

For immunofluorescent analysis, rehydrated tissue sections were subjected to heat-induced antigen retrieval in citrate buffer (pH 6) or Tris-EDTA buffer (pH 9), or proteolytic-induced antigen retrieval using proteinase K in Tris-EDTA-CaCl_2_ buffer (pH 8), washed and blocked with 1% fetal bovine serum (FBS, Biochrom, Berlin, Germany) in PBS for 30 min. Blocked samples were incubated with primary antibodies (polyclonal cross-reactive rabbit anti-human CD3, Dako, Hamburg, Germany; monoclonal rat anti-mouse F4/80, in-house production; monoclonal rat anti-mouse CD4, eBioscience; polyclonal rabbit anti-mouse COX-2, abcam, Cambridge, UK; polyclonal rabbit anti-mouse MPO, abcam; polyclonal goat anti-mouse IgA, SouthernBiotech, Birmingham, USA; polyclonal rabbit anti-mouse claudin (CLDN)1, LSBio, Seattle, USA; polyclonal rabbit anti-mouse CLDN4, LSBio; polyclonal rabbit anti-mouse occludin (OCLN), LSBio; polyclonal rabbit anti-mouse *zonula occludens* protein (ZO)1, LSBio) at a dilution of 1:50 to 1:1000 at room temperature (RT) for 2 h or at 4 °C overnight, respectively. After washing with PBS-T, slides were incubated with the corresponding secondary antibody (goat anti-rabbit IgG, carbocyanine (Cy)3-conjugated, Dianova, Hamburg, Germany; goat anti-rabbit IgG, biotinylated, Novex, Waltham, USA; donkey anti-rat IgG, biotinylated, Jackson Immuno Research, West Grove, USA; rabbit anti-goat IgG, biotinylated, Jackson Immuno Research) at a dilution of 1:200 to 1:500 at RT for 1 h. In case of biotinylated secondary antibodies, slides were further incubated with a streptavidine-Cy3 conjugate (ExtrAvidin^®^-Cy3^™^; Sigma-Aldrich, St. Louis, USA) for 30 min at RT. Stained sections were washed and mounted with a 4′,6-diamidin-2-phenylindol (DAPI)-containing mounting medium (Fluoroshield^™^ with DAPI; Sigma-Aldrich). Pictures were taken and digitized using an image scanner (Zeiss AxioScan.Z1; Zeiss) and quantification was done with ImageJ software (Version 1.46r; Wayne Rasband, National Institutes of Health, USA). Signals for Cy3 and DAPI were measured in a defined region of interest (ROI) applying constant greyscale limits. Expression of the markers was presented as Cy3-positive area relative to the DAPI-positive area [%]. For each treatment group, two sections per animal were analysed.

### ELISA

A piece of the colon was transferred into a 1.5 ml-Eppendorf tube containing 1 ml of ice-cold homogenate buffer (PBS + 10% FBS (Biochrom) + cOmplete^TM^ protease inhibitor cocktail, EDTA-free (Roche Applied Science Mannheim, Germany)). A colon homogenate was prepared using an Ultra-Turrax^®^ (IKA, Staufen, Germany). The homogenate was centrifuged at 21,000 × g, 10 min, 4 °C and the supernatant was transferred into a new tube. Samples were stored at −80 °C until analysis. For detection of CXCL1/KC, IL-1β, and IL-10 by ELISA, the DuoSet^®^ ELISA Mouse CXCL1/KC (R&D Systems, Wiesbaden-Nordenstadt, Germany), Mouse IL-1β ELISA Ready-SET-Go!^®^ and Mouse IL-10 ELISA Ready-SET-Go!^®^ (eBioscience, Frankfurt am Main, Germany) were used according to the manufacturer’s instructions. Supernatants were used undiluted. The concentration was quantified by measuring the optical density (OD) at 450 nm using a conventional microplate reader (Tecan Safire2; Tecan Group, Männedorf, Switzerland).

### Data availability statement

All data generated or analysed during this study are included in this published article.
